# The Effect of the Number of Sets on Power Output for Different Loads

**DOI:** 10.1515/hukin-2015-0043

**Published:** 2015-07-10

**Authors:** Antonio J. Morales-Artacho, Paulino Padial, Amador García-Ramos, Belén Feriche

**Affiliations:** 1 Deparment of Physical Education and Sport. Faculty of Sport Sciences, University of Granada.

**Keywords:** power training, power test, strength, velocity, postactivation potentiation, bench press

## Abstract

There is much debate concerning the optimal load (OL) for power training. The purpose of this study was to investigate the effect of the number of sets performed for a given load on mean power output (P_mean_). Fourteen physically active men performed 3 sets of 3 bench-press repetitions with 30, 40 and 50 kg. The highest mean power value (P_max_) across all loads and P_mean_ were compared when data were taken from the first set at each absolute load vs. from the best of three sets performed. P_mean_ increased from the first to the third set (from 5.99 ± 0.81 to 6.16 ± 0.96 W·kg^−1^, p = 0.017), resulting in a main effect of the set number (p < 0.05). At the 30 kg load P_mean_ increased from the first to the third set (from 6.01 ± 0.75 to 6.35 ± 0.85 W·kg^−1^; p < 0.01). No significant effect was observed at 40 and 50 kg loads (p > 0.05). P_max_ and velocity were significantly affected by the method employed to determine P_mean_ at each load (p < 0.05). These results show a positive effect of the number of sets per load on P_mean_, affecting P_max_, OL and potentially power training prescription.

## Introduction

The capacity to generate maximal upper body power has been suggested to be a determinant factor for success in sport activities requiring an optimal relationship between force and velocity. These may include throwing an object or explosively overcoming an external resistance (i.e. an opponent in judo or water in swimming) (Baker and Newton, 2005).

Maximal power is determined by optimal levels of force and velocity (Argus et al., 2013). Consequently, numerous studies have attempted to establish the load at which power is maximally developed (optimal load, OL) (Jandacka and Vaverka, 2008; Argus et al., 2013). Power-load and force-velocity curves are often based on a gradually increasing-load test while performing muscular tasks at maximal velocity (McMaster et al., 2014). Nevertheless, there is no general consensus in the literature regarding the exact procedure to obtain the force-velocity relationship during multijoint movements. Specifically, it is debatable how many cumulative exercise sets per load should be accomplished to accurately determine the OL, with numbers from 1 to 3 sets previously reported (Izquierdo et al., 2002; Lawton et al., 2006; Limonta and Sacchi, 2010; Marques et al., 2007; Sánchez-Medina et al., 2010; Thomas et al., 2007; Jandacka and Uchytil, 2011).

Earlier research has described the effect of preceding muscular activity on subsequent neuromuscular performance (Tillin and Bishop, 2009; Froyd et al., 2013; Wilson et al., 2013) as improvements in actions such as throwing, jumping, or hitting a mobile object (Gossen and Sale, 2000; Sale, 2002). Potential physiological mechanisms include increment in the number of actomyosin bridges, stimulation of the central nervous system (Rixon et al., 2007) and enhanced recruitment of motor units following prior activity (Güllich and Schmidtbleicher, 1996). Consequently, an upward and rightward shift in the force-velocity curve, induced by potentiation, has been previously proposed (Sale, 2002). It could potentially affect OL determination when performing multiple sets per load in order to obtain the force-velocity profile.

Among other factors, it seems that stimulus variability (Baudry and Duchateau, 2004; Gossen and Sale, 2000), training experience (Wilson et al., 2013), the muscle group examined (Pääsuke et al., 2007) or the time elapsed between the conditioning activity and the task (Gossen and Sale, 2000) may affect the balance between potentiation and fatigue. Previous research suggests that specificity of the conditioning activity is important when inducing post activation potentiation (Bonitch-Domínguez et al., 2010). Also, conditioning stimulus involving moderate intensities and including multiple sets has been proposed to be more efficient than heavy conditioning exercises (Wilson et al., 2013) in trained participants. All these factors could possibly affect power output and OL determination when performing various sets per load during the force-velocity curve protocol. It was thus hypothesised that power output performance and the OL could be significantly affected by the number of sets accomplished for a given load during the force-velocity curve.

Therefore, the purpose of the present study was to examine the effects of multiple sets of bench press repetitions conducted at submaximal progressive loads on the maximum power output and OL determination.

## Material and Methods

### Participants

Fourteen healthy, physically active male students were recruited from the student community of Granada University. All participants had a minimum of 3 years of experience in strength training. Age, body mass and height were 22.73 ± 3.97 years, 77.26 ± 9.21 kg and 178.93 ± 5.08 cm, respectively. All participants read and signed an informed consent form. The study protocol adhered to the tenets of the Declaration of Helsinki and was approved by the Ethics Committee of the Granada University.

### Measures

An intra-group repeated measures design was employed to examine the influence of performing three sets versus only one set, for a given absolute load, on power output. A linear position transducer was used to measure concentric-phase barbell velocity during the bench press exercise. The absolute workloads tested were 30, 40 and 50 kg.

### Procedures

**Figure 1:**
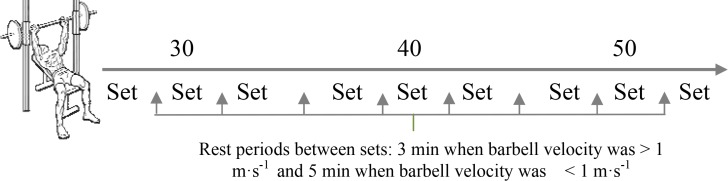
The procedure carried out to assess the force-velocity profile and power output

In a single visit to the laboratory and following a standard warm-up, participants underwent a force-velocity profile assessment. It comprised 3 sets of 2–3 concentric-only bench-press repetitions with three increasing absolute loads (Figure 1). To avoid forward and backwards barbell displacements, testing was performed in a Smith machine (Technogym, Italy).

In general, the intensity linked to maximal power development is approximately 30% of the maximal isometric force (MIF) (Cormie et al., 2011). For the bench press exercise, the literature describes a range of 30–60% 1RM (35–70 kg) as optimal for power development (McMaster et al., 2014; Castillo et al., 2011; Newton et al., 1997). Estimations of MIF for our participants (94.33 ± 16.53 kg) were made from the force–velocity curve. It provided evidence for the presence of the three absolute loads (30, 40 and 50 kg) within the intensity range mentioned above (27 to 65% of the MIF).

Rest periods between sets were 3 min when barbell velocities were ≥ 1m·s^−1^ and 5 min when the barbell velocity was slower than 1 m·s^−1^. Participants were instructed and verbally encouraged to achieve maximal velocity of the bar during each repetition. To ensure exclusive concentric-phase work, the barbell was kept still for 2 s pauses in the starting position (velocity = 0) 3–5 cm above the chest.

Mechanical variables were obtained using a Real Power Pro electronic linear transducer (Globus Italia connected to a Tesys 400 system) and the ErgoSystem 8.5 software. The system was fixed to the bar using a clip such that the cable would be vertically displaced and track the position of the bar during its movement (1000 Hz).

For each repetition, applied force (*F*), velocity (*V*) and power values were obtained. Data were expressed relatively to body weight (W·kg^−1^). A single repetition was selected as the best for each set and load, corresponding to the highest mean power and designated as the mean power (P_mean_). Maximal power (P_max_) was taken as the highest mean power recorded throughout the entire test. Then, P_mean_ for each load and P_max_ of the test were obtained to compare the first set to the best of the 3 sets accomplished per each absolute load. The optimal load (OL) was defined as the mechanical load (kg) at which P_max_ was achieved.

### Statistical Analysis

Data are expressed as mean ± standard deviation. Frequency distributions were assessed using the Shapiro-Wilk test. A two-way repeated measures ANOVA was employed to assess the influence of the set number (1^st^ vs. 2^nd^ vs. 3^rd^) and load (30 vs. 40 vs. 50 kg) on power output. When an interaction or main effect occurred, pairwise comparisons were made using Bonferroni post hoc analysis. Greenhouse-Geisser correction was selected when the Mauchly’s test of Sphericity was significant. The magnitude of the differences between both conditions was expressed as a standardized mean difference (Cohen’s d effect size; ES). The criteria to interpret the magnitude of the ES were as follows: <0.2 = trivial, 0.2–0.6 = small, 0.6–1.2 = moderate, 1.2–2.0 = large, 2–4.0 = very large and >4 = extra-large (Hopkins et al., 2009).

Paired t-tests were used to compare P_mean_ values achieved in the first set vs. the best of the 3 sets for each absolute load. Furthermore, paired t-tests were also used to compare the force and velocity values linked to each P_mean_ value selected. Significance was set at *p* ≤ 0.05 and all statistical analyses were performed in SPSS (v. 19).

## Results

There was a main effect of the set number (ANOVA, *p* = 0.028; F_exp_ = 4.10; 2, 26 *df*) due to an increase in P_mean_ from the first to the third set (5.99 ± 0.81 vs. 6.16 ± 0.96, *p* = 0.017, respectively). Pairwise comparisons revealed that these differences were due to an increase in P_mean_ from the first to the third set at the 30 kg load (6.01 ± 0.75 to 6.35 ± 0.85 W·kg^−1^, respectively; *p* < 0.01). For the remaining workloads, no significant effects of the set number on P_mean_ were observed (*p* > 0.05).

There was a main effect of the load (ANOVA, *p* = 0.003; F_exp_ = 10.39; 1.3, 17.1 *df*) taking into account lower P_mean_ values with 50 kg compared to the 30 kg (*p* = 0.012) and 40 kg loads (*p* = 0.001). No differences were observed in P_mean_ values attained with 30 and 40 kg (*p* = 0.646). No set number by load interaction effect was shown (*p* = 0.249). Inter set and inter load comparisons are shown in Table 1.

At each load, significant differences were observed in P_mean_ when the first set vs. the best of the three sets was compared (t-test, *p* < 0.01; Table 2). P_mean_ was located in the 2.20 ± 0.77 set number for 30 kg, the 2.33 ± 0.72 set number for 40 kg and the 2.00 ± 0.78 set number for 50 kg.

P_max_ and mean velocity were also significantly affected by the method employed to determine P_mean_ for each load (*p* < 0.05; Table 3). Applied force and the OL linked to P_mean_ values are also shown in Table 3.

## Discussion

The main finding of the present study is that the number of sets conducted at a given load affects maximal power output and OL determination. Consequently, the three cumulative bench press sets influenced positively power output. Our results may be of interest for sports practitioners and scientists when testing and prescribing power strength training.

Post activation potentiation effects have previously been reported in vitro (Metger et al., 1989) and in vivo conditions (Sale, 2002). Training studies have shown acute improvements in power and explosive strength performance following protocols with moderate and heavy loads (Güllich and Schmidtbleicher, 1996; Young et al., 1998; Ferreira et al., 2012). However, to the best of our knowledge, this is the first study to directly address the practicality of post activation potentiation in the OL determination and power development testing. Our results showed a P_max_ improvement and a tendency for OL decrement when the highest power output was selected from the three sets per load instead of using only the first set.

The effects of post activation may be confounded by factors such as variations in the type of stimulus (Baudry and Duchateau, 2004; Gossen and Sale, 2000), the muscle group assessed (Pääsuke et al., 2007) or training experience of participants (Wilson et al., 2013). In line with previous studies (Bonitch-Domínguez et al., 2010), our results suggest that potentiation effects can be observed when using the same exercise and load as conditioning activity.

In addition, the rest period between conditioning activity and performance assessment is known to be important to allow optimal balance between fatigue and potentiation effects (Gossen and Sale, 2000). Previous investigations have attributed the lack of potentiation effects to insufficient rest periods for a given volume of conditioning work (Gossen and Sale, 2000; Chaouachi et al., 2011). Based on the velocity of barbell displacement, we adjusted rest periods so that participants rested longer (5 min) between sets when the capacity to develop force quickly (barbell velocity) decreased. However, and despite of the low volume of work performed (2 – 3 repetitions per set), no effect of the set number was observed at 40 and 50 kg loads, presumably due to fatigue accumulation (Table 1). Previous research has reported optimal potentiation effects in bench-press 7 min after 1RM (Ferreira et al., 2012), which might suggest that our rest periods were not long enough at higher workloads. Notwithstanding, significant P_mean_ increments were observed when all sets at each load were taken into account (Table 2).

Training experience is also known to be an important factor that may affect balance between a rest period and volume of work (Wilson et al., 2013; Tillin and Bishop, 2009). Our participants were moderately trained, however, no fatigue symptoms were reflected due to similar power output from the 1st to 3rd set at the 50 kg load. Hence, it seems that interactions among all these factors may determine the relationship between optimal volume, workload and recovery to induce an optimal potentiating effect.

In agreement with Sale’s (2002) hypothesis, our results showed an effect of the set number on P_mean_, inducing an upward and leftward shift in the power-load curve. This is supported by previous research which linked the efficiency of prior explosive exercise to subsequent increments in electrical muscle activity (Bigland-Ritchie et al., 1986; Gossen and Sale, 2000), likely due to higher calcium sensitivity (Sale, 2002). This observation establishes a need to define the ideal protocol to efficiently measure P_max_ and OL. These results add information to the existing void concerning power-load curve protocols, which have usually included one (Thomas et al., 2007) or two (Jandacka and Vaverka, 2008; Izquierdo et al., 2002) sets per load. When the best set vs. the first was selected, P_max_ was improved by ∼6%, and barbell velocity increased by ∼10%.

A few limitations of the present investigation should be taken into account when interpreting our results. The fact that testing procedures involved concentric-only bench press exercise (non-ballistic) may be responsible for underestimations of power performance. Cronin et al. (2003) reported greater velocity development, but not strength, when the bar was released compared with concentric-only bench press exercise. Notwithstanding, Frost et al. (2008) showed that when the lifting phase was limited to only positive work, the difference between ballistic (i.e. bench press thrown) and non-ballistic bench press exercises was not significant in loads above 30% 1RM. Nevertheless, although similar results would be expected during ballistic bench-press exercise, further research involving upper and lower body exercises is needed to firmly understand potentiation phenomenon in muscle power assessments.

In conclusion, our findings indicate an effect of the number of sets conducted at each load on power and velocity measurements. If only one set per load was performed, the training load linked to maximal power would be overestimated, affecting the barbell velocity as well. When we only considered the first set of bench press repetitions, P_max_ was underestimated and the associated load was overestimated by ∼8% (p < 0.05). By including two sets per load in the assessment protocol, we can avoid overestimating the OL and maximal power. This could have a significant impact on power training prescription and training induced adaptations.

## Figures and Tables

**Table 1:** Mean power outputs relative to body mass recorded for increasing numbers of bench press repetitions (intra-load) performed at increasing training loads (inter-load)

P_mean_ W·kg^−1^	30 kg	40 kg	50 kg	*P*_2_
1^st^ set	6.01 ± 0.75	6.21 ± 0.86	5.74 ± 0.92*^b^*	*0.002*
2^nd^ set	6.27 ± 0.78	6.22 ± 0.93	5.81 ± 1.03*^c^*	*0.008*
3^rd^ set	6.35 ± 0.85*	6.31 ± 0.99	5.82 ± 1.17*^b, c^*	*0.005*
*P_1_*	*0.005*	*0.505*	*0.666*	

Data expressed as observed mean values ± SD. P_mean_ = mean power; P_1_ = intra-load comparisons (ANOVA); P_2_= inter-load comparisons (ANOVA).

***a*** = *difference 30 versus 40 kg;*

***b*** = *difference 40 versus 50 kg;*

***c*** = *difference 30 versus 50 kg;*

* difference set 1 versus 3 (p = 0.017).

**Table 2:** Mean power output from the first and best set from each absolute load

		30 kg	40 kg	50 kg
1^st^ Set (W·kg^−1^)		6.01 ± 0.75	6.21 ± 0.86	5.74 ± 0.92
Best Set (W·kg^−1^)		6.46 ± 0.77	6.48 ± 0.90	5.97 ± 1.06
*p*		*<0.001*	*<0.001 *	*0.013*
95% CI	LL	−0.265	−0.171	−0.055
	UP	−0.630	−0.384	−0.394
ES		0.59	0.31	0.23

Data expressed as observed mean power values ± SD. 1^st^ Set = highest mean power value observed at the first set of each load; Best Set = highest mean power value achieved in all sets performed at each absolute loads; CI = confidence interval; lower limit (LL); upper limit (UP); p = probability error; ES = effect size.

**Table 3:** Maximal power and its associated variables obtained according to the protocol used

		
		P_max_ (W·kg^−1^)	V (m·s^−1^)	F (N)	Load (kg)
1st Set		6.28 ± 0.84	0.95 ± 0.12	6.00 ± 0.84	38.6 ± 5.3
Best Set		6.66 ± 0.89	1.05 ± 0.13	5.82 ± 0.92	35.7 ± 6.5
*p*		*<0.001*	*0.002*	0.361	*0.104*
CI 95%	LL	−0.530	−0.143	−0.23	−0.67
	UP	−0.226	−0.039	0.60	6.39
ES		0.44	0.81	−0.21	−0.49

Data expressed as observed values ± SD. P_max_ = mean maximal power output; V= velocity; F= force; Load = absolute load at which power output was achieved.

1st Set = first set performed at each load; Best Set= best set out of the three performed at each load; CI = confidence interval; lower limit (LL); upper limit (UP); p = probability error; ES = Effect Size.
